# A dataset of major and trace element composition in sediments of the Loire river basin (France), records covering 1778-2022

**DOI:** 10.1016/j.dib.2026.113162

**Published:** 2026-08-18

**Authors:** Louis Manière, Cécile Grosbois, Elie Dhivert, Leslie Mondamert, Jérôme Labanowski, Marc Desmet, Florentina Moatar, Michel Meybeck

**Affiliations:** a Université de Tours. UR 6293 GéHCO (GéoHydrosystèmes continentaux). F-37200 Tours, France; b AnthropoSed. F-39130 Etival, France; c Université de Poitiers. UMR CNRS 7285 IC2MP (Institut de Chimie des Milieux et Matériaux de Poitiers). F-86073 Poitiers, France; d Institut National de Recherche pour l’Agriculture, l’Alimentation et l’Environnement, Riverly, centre de Lyon-Grenoble auvergne-Rhône-Alpes, 69100, France; e Sorbonne Université-CNRS-EPHE, UMR 7619 METIS, Paris, France

**Keywords:** Surface sediment, Core, Riverbank, Chemical composition, Contaminant

## Abstract

This paper introduces a comprehensive database documenting major, metallic, metalloid and rare earth elements in sediments throughout the entire Loire Basin, one of the largest basin of western Europe, flowing to Atlantic ocean. This unique compilation integrates three complementary datasets dealing with sediment collected during 2010-2022 and cores represent concentrations ranging from the mid-19th century up to 2022, riverbank profiles and surface sediments collected in the mid 2010s. The sediment core dataset provides a detailed chronological record and includes sedimentological and geochemical analyses from five cores collected along the Loire River, eight cores from its major tributaries, and one from a neighbouring river (Vilaine). In total, 498 dated samples were analysed for 64 parameters offering insights into long-term environmental changes. To enhance the spatial coverage across the basin, the core-based analyses are supplemented by data from 112 sediment samples recovered in riverbank profiles. These samples were strategically collected in sections of the basin where obtaining sediment cores was not unfeasible. Furthermore, the dataset includes analyses of 91 surface sediment samples, which serve to characterize and locate potential present and past anthropogenic sources of trace elements, thereby providing context for sediment records. This rich and multi-faceted dataset offers a robust spatio-temporal record of contaminants driven by anthropogenic impacts. It is a valuable resource for various environmental research applications. Locally, it has already been successfully utilized to investigate the key drivers of contaminant transfer within the Loire Basin. Globally, the scale and comprehensive nature of this dataset make it particularly well-suited for cross-referencing and comparative studies with other European large-river basins. Currently, the spatial and temporal coverage is limited worldwide. However, this work can contribute to a better understanding of human-induced environmental changes across the continent. Finally, particularly within the European Union context, this dataset also serves an operational purpose. It offers in-depth contextualization for contaminant data collected and archived by stakeholders during regulatory surveys.

Specifications Table

**Table:** 

Subject	Earth & Environmental Sciences
Specific subject area	Major and trace elements concentrations in different types of sediments (surface, riverbanks and cores) of the Loire River and it is main tributaries (France, 1850-2022)
Type of data	Tables (.tab format), Figure Raw
Data collection	Data were collected on cores, river bank profiles and surface sediment samples across the Loire basin, its main tributaries and the Vilaine river by several campaigns from 2010 to 2022. Major and minor element concentrations were determined by ICP-OES (iCap 6500, Thermo Scientific), trace elements by ICP-MS (Thermo Elemental X7, Thermo Scientific), except for mercury (Hg), which was analyzed using a DMA-80 analyzer (Milestone). Total carbon (TC) and total sulfur (TS) were measured via O₂ combustion at 1350 °C using a SC 144-DRPC analyzer (Leco) as total organic carbon (TOC) after acid pre-treatment with HCl. Model ages, when available, are based on grainsize variations and ^137^Cs activities.
Data source location	Institution : University of Tours City: Tours Country : France GPS coordinates : −3.63,136,967,44.30,865,703,5.10,567,750,48.95,142,851
Data accessibility	Repository name: PANGAEA Data identification number: https://doi.pangaea.de/10.1594/PANGAEA.986591 Direct URL to data: https://doi.pangaea.de/10.1594/PANGAEA.986591
Related research article	Grosbois, C.; Dhivert, E.; Rodrigues, S.; Le Guern, J.; Gaspéri, J.; Mondamert, L.; Labanowski, J. Drivers of contaminated sediment dynamics over 80 years at a basin scale (Loire river basin, France): a multifactorial approach. Journal of Environmental Management, (2025), 394, 127,494, https://doi.org/10.1016/j.jenvman.2025.127494

## 1 Value of the Data

• This data can be integrated with comparable datasets from other river basins to enhance the understanding of anthropogenic impacts at broader spatial scales and to document long-term environmental trajectories in large fluvial systems.
• The dataset also represents an archive of various anthropogenic influences within France’s largest basin among major European rivers. In addition, several deep sediment cores provide a reliable geochemical baseline, capturing pre-industrial levels for a wide range of trace elements [1].
• The dataset serves as an inventory of trace element concentrations associated with major urban, industrial and mining activities at a given reference period. It thus constitutes an essential reference for future temporal comparison, including assessments of changes in anthropogenic emissions and the effectiveness of environmental and/or regulatory measures. This scientific approach with sediment cores offers an in-depth contextualization across time and space, extending beyond the scope of regulatory surveys. Furthermore, this dataset complements the contaminants database in surface sediments maintained by river stakeholders (Naiades - https://naiades.eaufrance.fr).
• Most of the core samples are physically stored at the Géohydrosystèmes Continentaux research unit of the University of Tours (France) and registered within the French National Centre for Scientific Research (CNRS) core repository (https://www.cybercarotheque.fr). This long-term preservation ensures that additional analyses can be carried out in the future, such as using new analytical techniques and/or parameters according to storage and stability conditions.

## 2 Background

This detailed dataset supports original research studies on metal, metalloïd and rare earth elements in sediments of the Loire basin [[2], [3], [4], [5], [6]]. These river sediments databases are progressively being developed to analyse complex temporality of contaminant transport according to hydrosedimentary processes, industrial changes and river management operations at a basin scale (i.e. [7] for the Rhône river, [8] for the Saint-Charles river, [9] for the Ohre river] and to compare large rivers with various land-use historical specificities [6].

This joint investigation of sediment cores, river bank profiles and surface sediments was motivated to provide a comprehensive and complementary assessment of sediment sources and dynamics but also associated environmental changes over >50 years in the Loire river and its main tributaries. Beyond all these aims, this dataset was also designed to assess how station settling conditions and the sedimentary cascade could influence trace element transfers at a basin scale [[2], [10]]

## 3 Data Description

**Fig. 1:**
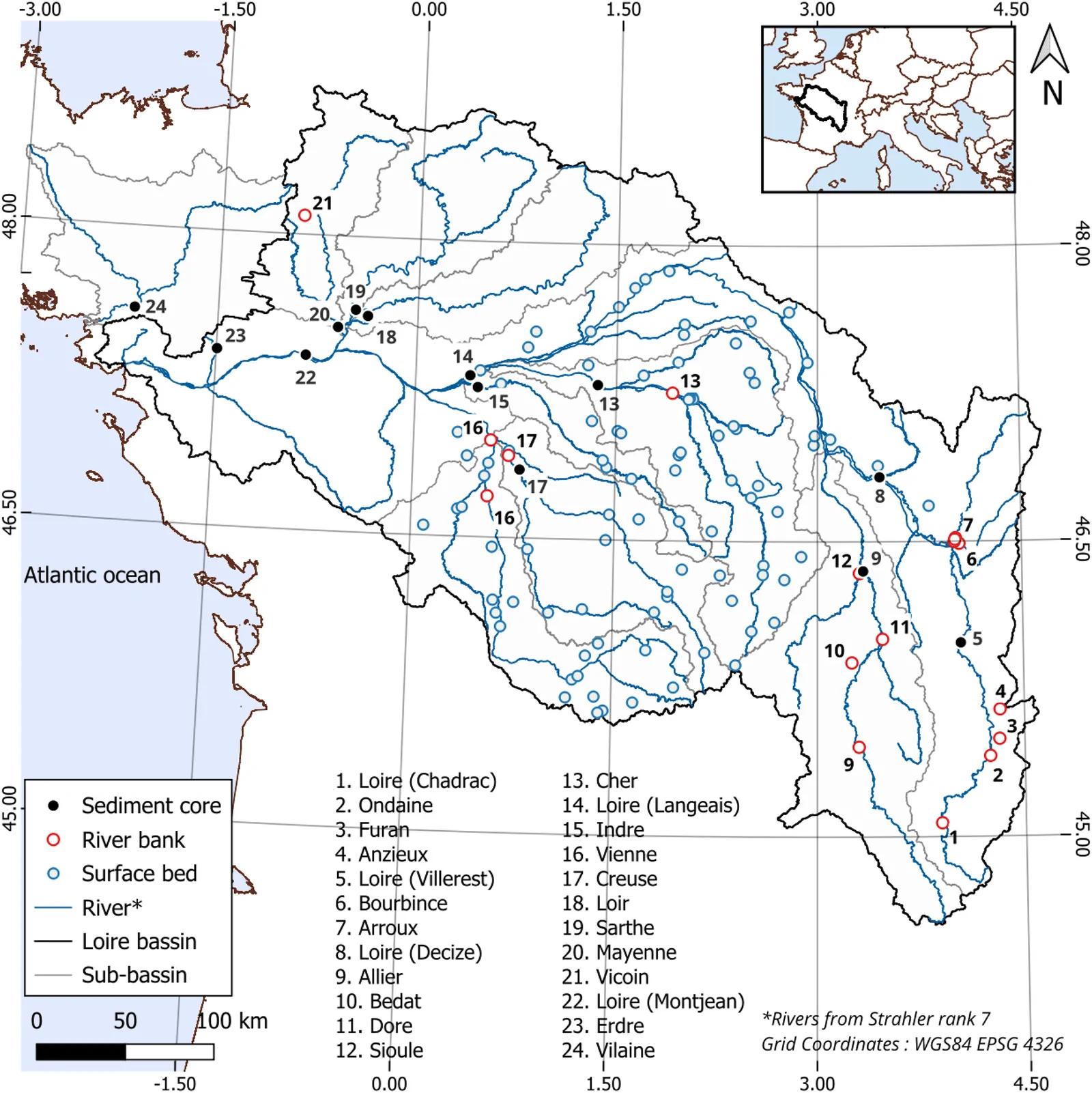
Localisation of the samples (sediment cores, sediment bank profiles and surface sediments, associated to their respective name stations in the Loire main stem and tributaries) in the Loire basin.

The dataset is organized by sediment sample type (core, riverbank profiles and surface sediments). Analysed sedimentological and geochemical parameters are almost the same except the lack of grain-size distribution for surface sediments and radioisotopes for riverbank and surface sediments. The spatial distribution extends along the main stem of the Loire river from upstream to downstream, as well as along its main tributaries and the Vilaine River (Fig. 1).

The dataset is stored in PANGAEA repository under the CC-BY license [11]. The dataset is a bundle of three individual datasets [[13], [14], [15]] composed with delimited text file based on PANGAEA sediment sample templates:

• The core, riverbank and surface sediment bundle: https://doi.pangaea.de/10.1594/PANGAEA.986591 [12]

All the dataset files have the same structure formatted by PANGAEA with the data description that include all the campaigns, parameters and events metadata, then the analysis results.

## 4 Experimental Design, Materials and Methods

### 4.1 Sediment core sampling method

The choice of each coring station was based on geographical and geomorphological criteria, with the objective of integrating the contribution of the widest possible range of potential upstream contamination sources. Therefore, stations were located as far downstream as possible from any major local contamination source (e.g. wastewater treatment plants, urban areas, mining or industrial activities), so that the specific geochemical signal of these local sources would not mask the contributions from other diffuse contamination sources. Another key criterion was the selection of settling environments that had undergone minimal disturbance or alteration over recent decades, in order to recover sedimentary cores as long as possible with coherent stratigraphy and potentially continuous sedimentation. For each selected station, a diachronic analysis was conducted using hydrological records (www.hydro.eaufrance.fr), historical maps, and orthophotographs covering several periods since the 19th century (https://remonterletemps.ign.fr).

Coring techniques were adapted to the specific conditions of each station (Table 1) to minimize disturbance of sedimentary layers. For underwater sampling in secondary channels and dam reservoirs (Loire at Villerest and Decize; Loir, Sarthe, Erdre and Vilaine stations), sediment cores were collected using a UWITEC corer equipped with a 90-mm-diameter PVC liner. At these locations, core compaction was minimal, limited to a few centimeters. Cores were stored horizontally at 4 °C for 24 to 48 hours prior to laboratory processing where they were opened, photographed, described and subsampled at 2 cm intervals and/or according to sedimentary transitions.

**Table 1:** Location and sampling characteristics of the sediment cores, from up- to downstream.

Sampling river ID	River basin @station Kilometric point KP	WGS84 coordinate system	Min - Max interannual average of monthly specific discharge (L/s/km^2^)	Deposition environment	Sampling device	Total core lenght (cm)	Sample number (ratio of analyzed / total samples)	Age model range
**The Loire stations**								
**5**	**Loire** **@**Villerest KP 266	4.0463943; 45.9850169	n.a.	Dam reservoir	Under water, Uwitec device	154	77 (100%)	1984–2012
**8**	**Loire** **@**Decize UWC KP 420	3.4597455; 46.8270107	n.a.	Semi-active channel, right bank	Under water, Uwitec device	160	80 (100%)	1850–2012
**8**	**Loire** **@**Decize RBC KP 420	3.4597455; 46.8270107	n.a.	River bank channel	Percusion corer	202	96 (100%)	1778–2012
**14**	**Loire @**Langeais KP 785	0.4068747; 47.3175409	2.3–12.6	Semi-active channel, left bank	stainless-steel peat corer	110	50 (96%)	1975–2018
**22**	**Loire** **@**Montjean s/L KP 891	−0.8287084; 47.3881254	2.3–13.9	River bank at the most downstream end of the island	Percusion corer	230	39 (100%)	1900s-2010s
**The Loire sub basin stations**								
**9**	**Allier** @Contigny	3.336054; 46.349515	3.5–15.2	Semi-active channel	Stainless-steel peat corer	100	49 (78%)	1940–2018
**13**	**Cher** @St Aignan	1.3617183; 47.2848314	1.5–11.5	Semi-active channel, right bank	Stainless-steel peat corer	50	22 (37%)	2005–2018
**17**	**Creuse** @Plaine des Patières	0.7938656; 46.8464601	2.4–18.0	Semi-active channel	Stainless-steel peat corer	36	9 (100%)	1975–2019
**15**	**Indre** @Azay le R.	0.4656836; 47.2591679	1.1–11.0	In the main channel, going through a castle moat,	Stainless-steel peat corer	100	49 (51%)	1970–2019
**20**	**Mayenne** @Montreuil-Juigné	−0.5969406; 47.5366277	1.9–21.6	Secondary active channel	Stainless-steel peat corer	160	53 (9%)	n.v.
**18**	**Loir** @Montreuil s/Loir	−0.3756389; 47.5990442	1.5–7.6	Secondary active channel	Under water, Uwitec device	80	40 (45%)	1980–2018
**19**	**Sarthe** @Porte Bise	−0.4682898; 47.6276627	1.9–13.2	Secondary active channel	Under water, Uwitec device	84	42 (69%)	1985–2018
**23**	**Erdre** @La Poupinière	−1.4943912; 47.3976436	n.a.	Dam reservoir	Under water, Uwitec device	109	11 (48%)	1950–2018
**Outside the Loire basin**								
**24**	**Vilaine** @Cran	−2.1281628; 47.5827752	n.a.	Dam reservoir	Under water, Uwitec device	94	15 (33%)	1956–2018

At stations located in semi-active channels connected to the main river for at least 8 to 9 months per hydrological year (Loire at Langeais; Allier, Cher, Creuse, Indre and Mayenne), coring was conducted using two stainless-steel peat corers, each 50 cm in length. This approach allows sediment recovery at true depth without compaction. The two corers were deployed simultaneously and in close proximity, resulting in the collection of up to eight cores per station: four cores from the 0–50 cm interval and, four from the 50–100 cm interval. Following detailed correlation of sedimentological features, each 50 cm sequence was subsampled at 2 cm resolution and/or following sedimentary transitions. Corresponding subsamples from parallel cores were homogenized in the field to ensure sufficient material for subsequent analyses. A field laboratory was therefore established at these sites to allow on-site core description, photography, subsampling, and packaging. Samples were preserved at 4 °C for 24 to 48 hours before laboratory processing. Sedimentary transitions and distinct event layers (e.g. coarse deposits, organic remains such as leaves or roots, and/or reworked sediments) were described and sampled separately. Throughout both field and laboratory operations, all equipment in contact with sediments was systematically rinsed with acidified deionized water (HNO₃ 30%) to prevent contamination.

For riverbank stations (Loire at Decize and Montjean), an Eijkelkamp percussion corer was used. Only sediment from the central portion of the core was retained and sliced into 5 cm intervals using a stainless-steel knife, which was cleaned with acidified deionized water (HNO₃ 30%) between each sampling layer. Samples were stored in plastic bags at 4 °C prior to laboratory processing. The Montjean record is composed of two coring campaigns (49MTJ09 and 49MTJ22): the initial core was collected in 2009, and a subsequent campaign in 2022 completed the archive with sediments from the 2010s (Table 1).

### 4.2 Riverbank profile sampling method

Riverbank profiles were collected using a PVC shovel rinsed with acidified water (HNO₃ 30%).

The 2010 SPAL10 and the 2013 METORG1 riverbank profile samples campaigns focused on a site selection downstream of major mining districts and industrialized zones. Sediments are collected specifically in the outer part of meanders, after refreshing the erosion surface. Two sampling protocols were applied:

• sediments were collected with continuous 20 cm vertical increments in visually homogeneous banks
• when distinct sediment layer in the riverbank showed visible stratification and/or variable colors and/or grain-size, it was sampled individually

The 2018 MetOrg3000 campaign also presented riverbank profiles sampled at stations where conditions were unsuitable for sediment coring, either due to the absence of representative depositional environments or to the abundance of coarse-grained deposits (Table 2). This was applied to the Arroux, Bourbince and Vienne sub-basins. For all these stations, a minimum of six samples per profile was collected using a PVC shovel in fresh bank surfaces in active erosion areas. Bank heights at these sites ranged from 70 cm to over 200 cm.

**Table 2:** Location and sampling characteristics of the sediment bank profiles.

Sampling river ID	River basin year	WGS84 coordinate system	Total bank height (cm)	Sample number
**SPAL10 campaign**				
6	Bourbince, 2010	46.4984150; 4.0065732	250	5
7	Arroux, 2010	46.5095135; 4.0112127	140	8
3	Furan, 2010	45.4953418; 4.3187611	275	14
16	Vienne, 2010	46.7086682; 0.5591780	165	7
17	Creuse, 2010	46.9166954; 0.7092504	175	12
13	Cher, 2010	47.2502997; 1.9202726	190	6
**METORG1 campaign**				
2	Ondaine, 2013	45.4087403; 4.2512974	120	6
12	Sioule, 2013	46.3386247; 3.3086723	80	4
1	Loire, 2013	45.0698537; 3.8990409	400	8
9	Allier, 2013	45.4565119; 3.3024805	100	5
10	Bedat, 2013	45.8847731; 3.2517701	100	5
4	Anzieux, 2013	45.6445084; 4.3241108	80	4
21	Vicoin, 2013	48.0948923; −0.8863151	120	6
11	Dore, 2013	46.0040896; 3.4759214	180	9
**METORG3000 campaign**				
7	Arroux, 2018	46.5171599; 4.0161941	90	5
6	Bourbince, 2018	46.4885989; 4.0418030	70	4
16	Vienne, 2018	46.9925007; 0.5743926	100	4

### 4.3 Surface sediment sampling method

All surface sediment samples were collected using a PVC shovel rinsed with acidified D.I. water (HNO₃ 30%).

The 2012 sampling campaign (METORG1) involved the collection of 91 riverbed and riverbank sediments downstream of various anthropogenic sources. These included areas historically impacted by former mining and associated activities, some industrial activities such as steel and coal-fired power plants (coal, U, Pb, Zn… [1]). The collected samples were used to identify some specific geochemical signatures associated with these anthropogenic activities.

### 4.4 Analyses

A total of 701 sediment samples were analysed, including 498 core samples, 112 samples from riverbank profiles and 91 surface samples.

All sediment samples were analyzed for major and trace element concentrations, as well as for total carbon and sulfur and organic carbon. Minor variations in analytical parameters were observed between sampling campaigns (Table 3).

**Table 3:** Parameters and their respective analytical methods by sediment sample type (core, riverbank and surface sediments).

Parameter	Method/Device	Core	Riverbank	Surface
Ag	ICP-MS Thermo Elemental X7, Thermo Scientific	X	(X)	X
As	ICP-MS Thermo Elemental X7, Thermo Scientific	X	X	X
Au			(X)	
B				
Ba	ICP-MS Thermo Elemental X7, Thermo Scientific	X	X	X
Be	ICP-MS Thermo Elemental X7, Thermo Scientific	X	X	X
Bi	ICP-MS Thermo Elemental X7, Thermo Scientific	X	X	X
Cd	ICP-MS Thermo Elemental X7, Thermo Scientific	X	X	X
Ce	ICP-MS Thermo Elemental X7, Thermo Scientific	X	X	X
Co	ICP-MS Thermo Elemental X7, Thermo Scientific	X	X	X
Cr	ICP-MS Thermo Elemental X7, Thermo Scientific	X	X	X
Cs	ICP-MS Thermo Elemental X7, Thermo Scientific	X	X	X
Cu	ICP-MS Thermo Elemental X7, Thermo Scientific	X	X	X
Dy	ICP-MS Thermo Elemental X7, Thermo Scientific	X	X	X
Er	ICP-MS Thermo Elemental X7, Thermo Scientific	X	X	X
Eu	ICP-MS Thermo Elemental X7, Thermo Scientific	X	X	X
F				X
Ga	ICP-MS Thermo Elemental X7, Thermo Scientific	X	X	X
Gd	ICP-MS Thermo Elemental X7, Thermo Scientific	X	X	X
Ge	ICP-MS Thermo Elemental X7, Thermo Scientific	X	(X)	X
Hf	ICP-MS Thermo Elemental X7, Thermo Scientific	X	X	X
Hg	DMA-80 (Milestone)	X	X	X
Ho	ICP-MS Thermo Elemental X7, Thermo Scientific	X	X	X
In	ICP-MS Thermo Elemental X7, Thermo Scientific	X	(X)	X
La	ICP-MS Thermo Elemental X7, Thermo Scientific	X	X	X
Li				
Lu	ICP-MS Thermo Elemental X7, Thermo Scientific	X	X	X
Mo	ICP-MS Thermo Elemental X7, Thermo Scientific	X	X	X
Nb	ICP-MS Thermo Elemental X7, Thermo Scientific	X	X	X
Nd	ICP-MS Thermo Elemental X7, Thermo Scientific	X	X	X
Ni	ICP-MS Thermo Elemental X7, Thermo Scientific	X	X	X
Pb	ICP-MS Thermo Elemental X7, Thermo Scientific	X	X	X
Pr	ICP-MS Thermo Elemental X7, Thermo Scientific	X	X	X
Rb	ICP-MS Thermo Elemental X7, Thermo Scientific	X	X	X
Sb	ICP-MS Thermo Elemental X7, Thermo Scientific	X	X	X
Sc	ICP-OES ICap 6500, Thermo Scientific	X	X	X
Se			(X)	
Sm	ICP-MS Thermo Elemental X7, Thermo Scientific	X	X	X
Sn	ICP-MS Thermo Elemental X7, Thermo Scientific	X	X	X
Sr	ICP-MS Thermo Elemental X7, Thermo Scientific	X	X	X
Ta	ICP-MS Thermo Elemental X7, Thermo Scientific	X	X	X
Tb	ICP-MS Thermo Elemental X7, Thermo Scientific	X	X	X
Th	ICP-MS Thermo Elemental X7, Thermo Scientific	X	X	X
Tl			(X)	X
Tm	ICP-MS Thermo Elemental X7, Thermo Scientific	X	X	X
U	ICP-MS Thermo Elemental X7, Thermo Scientific	X	X	X
V	ICP-MS Thermo Elemental X7, Thermo Scientific	X	X	X
W	ICP-MS Thermo Elemental X7, Thermo Scientific	X	X	X
Y	ICP-MS Thermo Elemental X7, Thermo Scientific	X	X	X
Yb	ICP-MS Thermo Elemental X7, Thermo Scientific	X	X	X
Zn	ICP-MS Thermo Elemental X7, Thermo Scientific	X	X	X
Zr	ICP-MS Thermo Elemental X7, Thermo Scientific	X	X	X
SiO_2_	ICP-OES ICap 6500, Thermo Scientific	X	X	X
Al_2_O_3_	ICP-OES ICap 6500, Thermo Scientific	X	X	X
Fe_2_O_3_	ICP-OES ICap 6500, Thermo Scientific	X	X	X
MnO	ICP-OES ICap 6500, Thermo Scientific	X	X	X
MgO	ICP-OES ICap 6500, Thermo Scientific	X	X	X
CaO	ICP-OES ICap 6500, Thermo Scientific	X	X	X
Na_2_O	ICP-OES ICap 6500, Thermo Scientific	X	X	X
K_2_O	ICP-OES ICap 6500, Thermo Scientific	X	X	X
TiO_2_	ICP-OES ICap 6500, Thermo Scientific	X	X	X
P_2_O_5_	ICP-OES ICap 6500, Thermo Scientific	X	X	X
LOI	Gravimetric analysis at 1020 °C (1040 °C for carbonates)	X	X	X
TC	O_2_ flow combustion at 1350 °C with a SC 144-DRPC (Leco)	X	(X)	X
TS	O_2_ flow combustion at 1350 °C with a SC 144-DRPC (Leco)	X	(X)	X
TOC	O_2_ flow combustion at 1350 °C with a SC 144-DRPC (Leco) after acid attack	X	X	X
Size < 63 µm	Mastersizer 2000, Malvern	X	(X)	
Size D10	Mastersizer 2000, Malvern	X	(X)	
Size D50	Mastersizer 2000, Malvern	X	(X)	
Size D90	Mastersizer 2000, Malvern	X	(X)	

(X) only available for some sampling campaigns.

To minimize grain-size effects on geochemical parameters, all analyses were carried out on the <63 µm sediment fraction, obtained by sieving through disposable meshes following the protocol described in [4]. An exception was applied to the Decize core, where samples from the 0–160 cm interval were analyzed on the bulk fine fraction, as the median grain size remained below 63 µm.

All major, minor, and trace element determinations were performed at the national analytical facility SARM-CRPG (Nancy, France). Samples were prepared for LiBO₂–Li₂B₄O₇ fusion in a tunnel oven, followed by acid dissolution in HNO₃. Major and minor element concentrations were measured by ICP-OES (iCap 6500, Thermo Scientific), while trace elements were quantified by ICP-MS (Thermo Elemental X7, Thermo Scientific), except for mercury (Hg), analyzed using a DMA-80 analyzer (Milestone). Total carbon (TC) and sulfur (TS), total organic carbon (TOC) contents were determined by oxygen combustion at 1350 °C using a SC 144-DRPC analyzer (Leco), TOC measured after acid pre-treatment with HCl. Analytical uncertainties were lower than 1 % for major elements and within 10 % for trace elements. Detection limits for each parameter are also specified in the corresponding data files, together with the device used.

Riverbank profile samples collected during the SPAL10 campaign (outcrop dataset) were also analyzed at SARM-CRPG using previous analytical facilities. Consequently, detection limits differ for these samples and are reported individually in the associated datasets.

For age-model determination, each stratigraphic level of the METORG3000 sediment cores was subdivided into four fractions for grain-size distribution, water content, apparent bulk density and radionucleide analyses. These 3 first types of analyses were carried out on fresh bulk sediment. With the exception of the Montjean core (events 49MTJ09 and 49MTJ22), grain-size distributions were determined for each sediment level using a laser diffraction microgranulometer (Mastersizer 3000, Malvern).

For radionuclide measurements (¹³⁷Cs, ²⁴¹Am, ²¹⁰Pb, and ⁷Be for surface samples), sediments were oven-dried at 105 °C for 24 h, sieved to <2 mm, and homogenized by hand crushing. Gamma-ray spectrometry analyses were performed at the PANOPLY analytical platform (Paris, France; https://panoply-geops.lsce.ipsl.fr/index.php/en/).

### 4.5 Age model description

Age models were validated for 13 sediment cores. The core from the Mayenne sub-basin (49MAY18 event) was excluded due to discontinuities caused by several centimeter-thick organic layers containing plant remains. In three other sub-basins (Arroux - 71ARO18, Bourbince - 71BOU18 and Vienne - 37VIE18 events), although multiple sites were investigated, any station fully met coring criteria and only undated riverbank profiles were collected.

Absolute dating was performed using ^137^Cs radiometric analyses on about 50 g of <2 mm core layers, sealed in airtight plastic containers for 24 hour gamma counting. Measurements were carried out with very low-background, coaxial HP Ge N-type detectors (8000 channels), with regular calibration using internal soil and sediment standards, pure KCl samples, and IAEA reference materials (Soils 6, 135, and 375).

The ^137^Cs activity profiles identified three major events within sediment cores:

• 1950, marking the onset of thermonuclear weapons testing and the first detectable increase in ^137^Cs
• 1962/63, corresponding to the maximum of atmospheric fallout, supported by the detection of ^241^Am presence
• 1986, representing the Chernobyl nuclear accident

Grain-size variations were also used to identify important flood events at each station. They were evidenced by increasing grain-size and coarsest sediment layers and then correlated with well documented hydrological records from nearby gauging stations. Depending on the core and its associated depositional environment, these flood deposits provided supplementary chronological markers within the age model.

For all the sediment cores, the determination of the age–depth model assumes that sedimentation is continuous, with only minor mechanical erosion and transport of surface sediments except for identified flood deposits.

Mass accumulation rates (MAR, kg·m⁻²·year⁻¹) were calculated between each absolute chronological marker according to the Van Meter et al. [16] ‘s method. First, the dry mass (DM; kg·m⁻²) is calculated to be able to compare between cores with varying water-saturated sediment levels and to correct for coring-induced compaction as follow:

DM=(1−n).DS.Th

where n is the porosity (%) representing the water volume in a known volume of sediment, determined from the weight difference between wet and dry sediment; DS is the apparent dry sediment density (g·m⁻³) within the defined volume; and Th is the thickness of the sampled layer.

The MAR was then determined by dividing the cumulative dry mass between two layers (cum, kg·m⁻²) by the time interval (TI, years):

MAR=cum/TI

The age of sediments (Date) at level i is calculated from the top of the core to the first chronological marker:

$$Date_{i}=coringdate-cum_{i}/MAR$$

For sediment layers *j* older than the first chronological marker *i*, the following equation was applied:

$$Date_{j}=markerdate_{i}-\left(cum_{j}-cum_{i}\right)/MAR_{j}$$

More detailed descriptions of the age models can be found in previous publications [[2], [3], [4], [10]].

## Limitations

The uneven chronological coverage among the core age models is a limitation that should be considered. The period from the 1970s to the late 2010s is the best represented by the dated sediment cores. Five of the thirteen cores extend even back in time; notably, the Montjean-sur-Loire core, the most downstream site, reaches back to the early 1900s whereas the Decize cores extend even further, down to the late 1770s, thus predating the Industrial Revolution. The sedimentary matrix variability (mineralogy, grainsize and organic matter content), inherent in river environments, must also be considered to ensure accurate interpretation and meaningful data reuse.

## Ethics Statement

The authors have read and follow the ethical requirements for publication in Data in Brief and confirming that the current work does not involve human subjects, animal experiments, or any data collected from social media platforms.

## CRediT Author Statement

**Louis Manière:** Data curation (lead), Writing – original draft (equal); **Cécile Grosbois:** Conceptualization (lead), Methodology (lead), Formal analysis (equal), Investigation (lead), Data curation (equal), Writing – original draft (equal), Funding acquisition (equal), Project administration (equal); **Elie Dhivert:** Conceptualization (equal), Methodology (equal), Formal analysis (equal), Investigation (lead), Writing – review and editing (equal), Funding acquisition (equal), Project investigation (lead); **Leslie Mondamert:** Conceptualization (equal), Methodology (equal), Formal analysis (equal), Investigation (equal), Writing – review and editing (equal), Funding acquisition (equal), Project administration (equal); **Jérôme Labanowski:** Conceptualization (equal), Methodology (equal), Formal analysis (equal), Investigation (equal), Writing – review and editing (equal), Funding acquisition (equal), Project administration (equal); **Marc Desmet:** Conceptualization (equal), Methodology (supporting), Formal analysis (supporting), Investigation (supporting), Writing – review and editing (equal), Funding acquisition (equal), Project administration (equal); **Florentina Moatar:** Conceptualization (supporting), Formal analysis (supporting), Investigation (supporting), Writing – review and editing (equal), Funding acquisition (equal), Project administration (equal); **Michel Meybeck:** Conceptualization (supporting), Formal analysis (supporting), Investigation (supporting), Writing – review and editing (equal), Funding acquisition (supporting), Project administration (supporting).

## Declaration of generative AI and AI-assisted technologies in the manuscript preparation process

During the preparation of this work the authors used DeepL and Microsoft Copilot in order to improve writing in English, to rephrase sentences and for summarising. After using these tools/services, the authors reviewed and edited the content as needed and take full responsibility for the content of the published article.

## Data Availability

PANGAEASpatial and temporal variations of major and trace elements in sediments of the Loire River basin (France, 1850-2022) (Original data).
